# Personal resources for addressing the work demands of Ukrainian oncologists in stressful crisis situations

**DOI:** 10.1186/s12889-024-18315-1

**Published:** 2024-03-13

**Authors:** I Böckelmann, I Zavgorodnii, O Litovchenko, V Kapustnyk, M Krasnoselskyi, Beatrice Thielmann

**Affiliations:** 1 Institute of Occupational Medicine, Faculty of Medicine, Otto von Guericke University Magdeburg, Leipziger Str. 44, 39120 Magdeburg, Germany; 2 Department of Hygiene and Ecology № 2, Kharkiv National Medical University, Kharkiv, Ukraine; 3 Department of Internal and Occupational Diseases, Kharkiv National Medical University, Kharkiv, Ukraine; 4 Grigoryev Institute of Medical Radiology and Oncology, National Academy of Medical Sciences of Ukraine, Kharkiv, Ukraine

**Keywords:** Physicians, cancer patients, Coping, Mental health, Crises

## Abstract

**Background:**

Many work-related stresses are experienced by oncologists. Ukraine is currently undergoing numerous crises, including the SARS-CoV-2 pandemic and military conflicts, which represent stressful situations. The aim of this study was to explore the personal resources that Ukrainian oncologists use to cope with work demands in a situation featuring manifold crises. This study identified the ways in which people deal with stressful situations and the roles that they play in shaping the challenging situations that they encounter (work-related behavior) as well as how they cope with stress (stress management).

**Methods:**

Forty oncologists (18 men and 22 women) working in a clinic in Kharkiv (Ukraine) with an average age of 46.3 ± 13.37 years (ranging from 26 to 74 years) participated in this study. The occupational psychological survey consisted of the Work-Related Behavior and Experience Patterns (German: Arbeitsbezogenes Verhaltens- und Erlebensmuster, AVEM) questionnaire, which was developed by Schaarschmidt and Fischer, and the Differential Stress Inventory (DSI), which was developed by Léfevre and Kubinger.

**Results:**

65% of oncologists exhibited AVEM risk pattern A or B. No gender differences were observed with regard to the distribution of AVEM patterns. Women obtained significantly higher scores than did men on only one dimension: experience of social support (4.86 vs. 3.44; *p* = 0.045). When the DSI categories were differentiated by gender, no significant differences were observed. Spearman’s correlation analysis revealed a medium-sized correlation between perfection striving and palliative coping (ρ = 0.404).

**Conclusions:**

Few gender-based differences in work-related behaviors, experiences, and stress management strategies are evident among oncologists. AVEM risk patterns are more prevalent among Ukrainian oncologists than among comparable occupational groups, and interventions in the context of health management are recommended.

## Background

Oncologists experience various types of work-related stress. The medical field entails considerable demands due to rapid scientific developments in diagnostics and therapy as well as the increasing number of cancer cases, which is partially due to rapid population aging. Researchers have shown that oncologists face life-and-death decisions with respect to their cancer patients [1]. This constant need to manage serious cancers, which are associated with limited and sometimes hopeless curative outcomes, alongside excessively long working hours, increased administrative burdens, limited autonomy in terms of daily tasks, and numerous electronic documentation requirements, appears to render oncologists more vulnerable to impaired mental health. These working conditions can also be observed among Ukrainian oncologists [1]. The SARS-CoV-2 pandemic and the military conflict in Ukraine represent additional stressors among Ukrainian oncologists. High levels of psychological stress can lead to mental health impairments such as sleep disorders, burnout, or depression [2–5].

Physicians often pay little attention to their own health and well-being [6, 7]. However, physicians’ health and well-being must be maintained at a high level if they are to be able to provide treatment to cancer patients. The effects of numerous work stresses on an individual depend on the type and amount of self-attention exhibited by those individuals as well as their internal evaluation and processing of stressful situations [8]. The way in which a stressful situation is addressed can contribute to the development of health impairments. Optimism, positive psychology, self-efficacy expectancy, resilience, and social support are important health resources that can promote the ability to cope with work-related stressors successfully [8–10].

The enhancement of employees’ personal resources may be beneficial with respect to their mental health [11]. Personal resources refer to aspects of the self that are generally linked to resiliency and pertain to individuals’ sense of their own ability to perceive, control and influence their environment successfully [12]. Personal resources are fundamental components of individual adaptability. Individuals who learn how to respond to unfavorable, stressful situations in an optimistic manner exhibit greater persistence, which is a requirement for successful adaptation [13]. Personal resources play an important role in the job demands-resources model [14, 15] since, alongside job demands and job resources, they contribute to explaining variance in exhaustion and work engagement [11].

When diagnosing occupational stress, a symptom-oriented approach is usually employed [16]. However, based on the current holistic concepts of mental health, one should not merely detect impairments and complaints. The salutogenetic approach [17] entails examining why people remain healthy despite experiencing numerous both everyday and occupational stresses, risk factors for chronic diseases, and previous and ongoing critical life events. Antonovsky’s model locates the “sense of coherence”, which features three components (meaningfulness, manageability and comprehensibility), at the core of the development of health, and this notion can also be transferred to work. According to the salutogenetic model, health should be understood not as a fixed and rigid state but rather as the goal of a complex process. Risk and protective factors work together [17]. Therefore, within the framework of salutogenetic health promotion, coping potentials are strengthened, and (company) framework conditions are established to preserve the health of employees [17]. In this context, it is beneficial to investigate work-related behavioral and experiential patterns and/or individual stress management strategies [18, 19]. Work-related behavior and experience patterns are personality-specific styles that are used to address work and professional requirements [20]. This factor is viewed as a personal resource that can be used early in the process of addressing stressful situations. This way of dealing with work demands is based on the three areas mentioned above, i.e., engagement with work, emotional resilience and work-related emotions. Each of these three aspects has particular relevance for statements concerning an individual’s health. Understanding these patterns and strategies can lead to improvements in targeted intervention measures as well as the training and coaching of employees [18, 19].

## Methods

### Aim

The aim of this study was to explore the personal resources used by Ukrainian oncologists to address work demands in a situation featuring manifold crises, including the SARS-CoV-2 pandemic and military conflicts. We identified the ways in which oncologistsdeal with stressful situations and the roles that they play in shaping the challenging situations that they encounter (work-related behavior) as well as how they cope with stress (stress management). An assessment of the personal resources that Ukrainian oncologists use to cope with work demands is performed, as these resources play an important role in mental health. This study also explores the work-related behaviors and coping styles of oncologists in stressful situations with respect to gender groups.

### Setting

Forty oncologists (18 males and 22 females) with an average age of 46.3 ± 13.37 years (ranging from 26 to 74 years) working in a clinic in Kharkiv (Ukraine) voluntarily participated in an occupational psychology survey. The mean age of the 18 male respondents was 47.4 ± 13.19 years. The mean age of the female respondents was lower (45.4 ± 13.76 years), but this difference was not significant (*p* = 0.697).

The inclusion criteria required respondents to have a medical practice that primarily focused on the field of oncology and to have at least one year of experience working as an oncological physician.

The physicians included in this research worked mainly in the field of oncology but could pursue different activities; accordingly, the sample included chemotherapists, oncologists, oncosurgeons, oncogynecologists, oncoradiologists, interventional radiologists, ENT oncologists, hematologists, and radiotherapists working in Kharkiv, Ukraine.

This online survey was administered from June to September 2022 via Google Forms. Prominent crisis situations at the time of the survey included the SARS-CoV-2 pandemic and the military conflict in Ukraine.

The survey was conducted in accordance with the bioethical requirements that were stipulated at meetings of the Committee on Ethics and Bioethics of Kharkiv National Medical University during the planning of the research (extract from Protocol No. 3 of August 28, 2020) as well as in accordance with the work plan for 2022 (extract from Protocol No. 3 of March 17, 2021).

Due to technical and organizational problems as well as the declaration of martial law and occurrence of active hostilities in Kharkov itself during this research period, the overall response rate could not be calculated precisely; however, it was estimated to be between 80 and 85%.

### Description of materials

The diagnostic instrument used in this study is the work psychology questionnaire *“Work-Related Behavior and Experience Patterns” (German: Arbeitsbezogenes Verhaltens- und Erlebensmuster, AVEM), which features 66 items according to Schaarschmidt and Fischer* [21]. This questionnaire captures behavior and experience in the context of perceived job demands. The instrument is used to identify the ways in which individuals respond to stressful situations and the roles that they play in shaping the challenging situations that they encounter. These patterns of behavior and experience, in turn, are key indicators of the levels of emotional health associated with the ways in which individuals relate to their work [20]. This method is based on the concept of resources [22] and used to assess personal resources in the context of work-related demands. This procedure provides important findings concerning the health-promoting/endangering behaviors associated with coping with stress based on eleven categories ranging across three areas (engagement with work, emotional resilience, and work-related emotions). The test involves the following 11 dimensions: Subjective importance of work (1), Work-related ambition (2), Willingness to work until exhausted (3), Striving for perfection (4), Distancing ability (5), Tendency to resignation in the face of failure (6), Proactive problem solving (7), Inner calm and balance (8), Experience of success at work (9), Satisfaction with life (10) and Experience of social support (11). Dimensions 1 to 5 describe engagement with work. Dimensions 5 to 8 represent emotional resilience, and dimensions 8 to 11 represent work-related emotions. Subsequently, AVEM patterns (A, B, G and S) for each respondent are obtained. With the help of a cluster analysis that follows the approach developed by Schaarschmidt & Fischer and the integration of the Vienna Test System (Schuhfried GmbH, Mödling, Austria), a stable and replicable classification of work-related behavior and experience patterns into four behavior and experience patterns (clusters) can be obtained. For each individual, the degree to which his or her individual profile matches the four reference profiles can be determined using cluster analysis. To calculate the profile adjustment, discriminant functions drawn from the discriminant analysis were used [20].

In *Pattern A*, the strongest values are associated with the importance of work, the willingness to work until exhausted, and striving for perfection, while the lowest value is associated with the ability to dissociate [23]. Individuals who exhibit *pattern B* (burnout) obtain low scores in the dimensions of engagement with work, especially with respect to the subjective importance of work and work-related ambition, as well as limited ability to dissociate and a high tendency to resign. These individuals exhibit very low levels of proactive problem solving, inner calm and balance. They also exhibit low levels of professional success, life satisfaction and social support. High levels of resignation, reduced motivation, decreased resilience to professional demands and negative emotions are common among individuals who exhibit *pattern B*. Both patterns A and B are AVEM risk patterns.

Individuals who exhibit *pattern G* engage in health-promoting behavior with respect to work. Such individuals are associated with clear but not excessive expressions in the dimensions associated with the area of commitment. Their work-related ambition is very strong. They exhibit a (healthy) capacity for detachment. Among all the patterns, *pattern G* is associated with the lowest tendency to resignation in the face of failure and the highest levels of proactive problem solving, inner calm and balance. These people possess emotional strength, professional success experience, and life satisfaction, and they feel that they have social support. People who exhibit *pattern S* (which indicates sparing at work) obtain the lowest scores regarding the significance of their work, work-related ambition, willingness to work until exhausted and striving for perfection. The ability to distance themselves from their work is notably strong among these individuals. The low tendency to resign in the face of failure exhibited by these individuals indicates that reduced commitment should not be interpreted as an expression of a resigned attitude. Their characteristic positive attitudes toward life are due to their high levels of inner calm and equilibrium as well as their perceived social support.

High stanine values indicate a higher level of the AVEM dimensions in question. With regard to the 11 AVEM scales used in the test, the split-half reliability was first calculated using the Spearman-Brown method, and the internal consistency was calculated using Cronbach’s alpha. Cronbach’s alpha ranged between 0.79 and 0.87.

*The Differential Stress Inventory (DSI) questionnaire developed by Lefèvre & Kubinger* as part of the Vienna Test System (Schuhfried Company, Mödling, Austria) was used as a diagnostic instrument to identify person-specific ways of addressing stress [18]. In this questionnaire, a total of 122 questions are summarized into factor-analyzed dimensions that cover four stress-relevant aspects: causes of stress, stress manifestation, coping, and stress stabilization. The questions ask respondents to report their experiences during the past 2–3 months [18]. With regard to the stress trigger dimension, three domains (worries about one’s life circumstances, difficulties arising from interactions with other people, and stressful everyday situations) are distinguished. The physiological, cognitive, and emotional levels are taken into account when assessing the stress manifestation dimension. A high score on this dimension highlights, among other things, the emergence of physical symptoms such as pain or the mental continuation of preoccupation with the stress, which can also limit the individual’s ability to act. The coping domain focuses on problem-related (instrumental) and emotion-related (palliative) strategies. High scores on this dimension indicate the presence of stress coping mechanisms, such as actively taking action or talking positively to oneself to cope with problems. Stress stabilization is understood as a reinforcer of internal intensifiers (mental continuation) and external intensifiers (illness gain), which can lead to the chronification of stress in cases featuring long-lasting stressful situations and the constant presence of stress.

High stanine values indicate a higher level of the DSI dimension. The reliability coefficients of the DSI dimension range between *r* = 0.72 and *r* = 0.94 (Cronbach’s alpha) depending on the scale or subscale in question.

### Statistical analysis

The sample that we were able to recruit for this research was small. The statistical processing and analysis of the data were performed using the software package IBM SPSS Statistics 26. First, frequency analyses were conducted with regard to the total sample with the inclusion of additional descriptive characteristic values, such as the mean (M) and standard deviation (SD) as well as the median, alongside the associated minimum (min) and maximum (max); subsequently, 95% confidence intervals were calculated. The variables were tested for normal distribution using the Shapiro‒Wilk test (which is employed when examining fewer than 50 samples). Furthermore, an exploratory analysis of gender differences was conducted. Most of the variables were not normally distributed; thus, nonparametric tests were chosen for this research. The significance level used to calculate the mean differences in the Mann‒Whitney test was 5%. With regard to the frequency analyses and cross-tabulations, the Pearson chi-square test was used for minimum expected frequencies > 5%, and Fisher’s exact test was used for minimum expected frequencies < 5%. The results of the AVEM and DSI questionnaires were analyzed to explore the sociodemographic data (age), and Spearman’s correlation analysis was used to quantify the associations.

## Results

### Sociodemographic data

The 18 men who participated in this research had been working for an average of 23.3 ± 13.3 years, and the 19 women respondents had slightly fewer years of employment (21.9 ± 13.5 years). However, these differences were not significant (*p* = 0.900). Regarding marital status, 6 (16.2%) respondents indicated that they were single, 22 (59.5%) indicated that they were married, 2 (5.4%) indicated that they were widowed and 7 (18.9%) indicated that they were divorced. Three participants did not answer this question. At the time of the survey, 24 (64.9%) oncologists were living in a stable partnership, while 13 (35.1%) respondents answered this question in the negative. The same three test subjects mentioned previously also left the question about a permanent partnership unanswered. The majority of oncologists lived with their children in the household (*n* = 26 (72.2%)), while for 10 respondents (27.8%), their children had already established their own household. One respondent indicated that their child had been evacuated from Ukraine at the time of this research. Twelve oncologists (33.3%) had a family member at home who needed care. The two gender groups did not exhibit significant differences in terms of marital status (*p* = 0.263), steady partnership (*p* = 0.495), children in the household (*p* = 0.717) or status as carers for family members in the household (*p* = 0.302).

### Patterns and dimensions of work-related behavior and experience

With regard to the total sample, the highest-scoring dimensions were all associated with the work engagement domain (Table 1). On average, all the other dimensions were within the reference range, with the individual maximum values being within an above-average range. The lowest-scoring dimension among the female oncologists was distancing ability. At the dimension level, the gender groups did not exhibited significant differences. In terms of social support, female oncologists obtained significantly higher stanine values than did male oncologists (4.86 vs. 3.44; *p* = 0.045). In addition, when all dimensions were taken into account, these stanine values were lowest among males. The three highest-scoring dimensions among men were work-related ambition, the subjective importance of work, and willingness to work until exhausted. Among female oncologists, the highest-scoring dimensions were striving for perfection, willingness to work until exhausted, and work-related ambition.

**Table 1:** The expression of the AVEM dimensions in the total sample and in both gender groups

AVEM	Men *n* = 18	Women *n* = 22	Total *n* = 40	p_Mann−Whitney_
	AV ± SD [stanine] median (min - max) 95% confidence interval			
Subjective importance of work	6.39 ± 1.461 7 (4–9) [5.66–7.12]	6.14 ± 1.833 7 (2–9) [5.32–6.95]	6.25 ± 1.660 7 (2–9)	0.861
Work-related ambition	6.44 ± 1.464 7 (3–8) [5.72–7.17	6.32 ± 1.359 6 (4–9) [5.72–6.92]	6.38 ± 1.390 6 (3–9)	0.619
Willingness to work until exhausted	6.33 ± 1.372 6 (5–9) [5.65–7.02]	6.36 ± 1.497 6 (4–9) [5.70–7.03]	6.35 ± 1.424 6 (4–9)	1.000
Striving for perfection	6.17 ± 1.855 6 (2–9) [5.24–7.09]	6.59 ± 1.501 7 (3–9) [5.93–7.26	6.40 ± 1.661 6 (2–9)	0.443
Distancing ability	4.50 ± 1.823 5 (1–7) [3.59–5.41]	4.00 ± 1.662 4 (1–8) [3.26–4.74]	4.22 ± 1.732 4 (1–8)	0.274
Tendency to resignation in the face of failure	5.83 ± 2.121 5.50 (2–9) [4.78–6.89]	5.32 ± 1.701 5.00 (2–9) [4.56–6.07]	5.55 ± 1.894 5 (2–9)	0.411
Proactive problem-solving	5.22 ± 2.557 5.00 (2–9) [3.95–6.49]	5.45 ± 1.503 6.00 (2–8) [4.79–6.12]	5.35 ± 2.020 5 (2–9)	0.600
Inner calm and balance	4.56 ± 1.464 5.00 (1–7) [3.83–5.28]	5.09 ± 1.630 5.00 (3–9) [4.37–5.81]	4.85 ± 1.562 5 (1–9)	0.492
Experience of success at work	4.28 ± 2.081 4.00 (1–8) [3.24–5.31]	4.32 ± 1.615 4.00 (1–7) [3.60–5.03]	4.30 ± 1.814 4 (1–8)	1.000
Satisfaction with life	4.50 ± 2.149 4.00 (1–9) [3.43–5.57]	4.23 ± 1.901 4.00 (1–7) [3.38–5.07]	4.35 ± 1.994 4 (1–9)	0.798
Experience of social support	3.44 ± 1.756 3.00 (1–7) [2.57–4.32]	4.86 ± 2.189 5.00 (1–8) [3.89–5.83]	4.22 ± 2.106 4 (1–8)	0.045

**Fig. 1:**
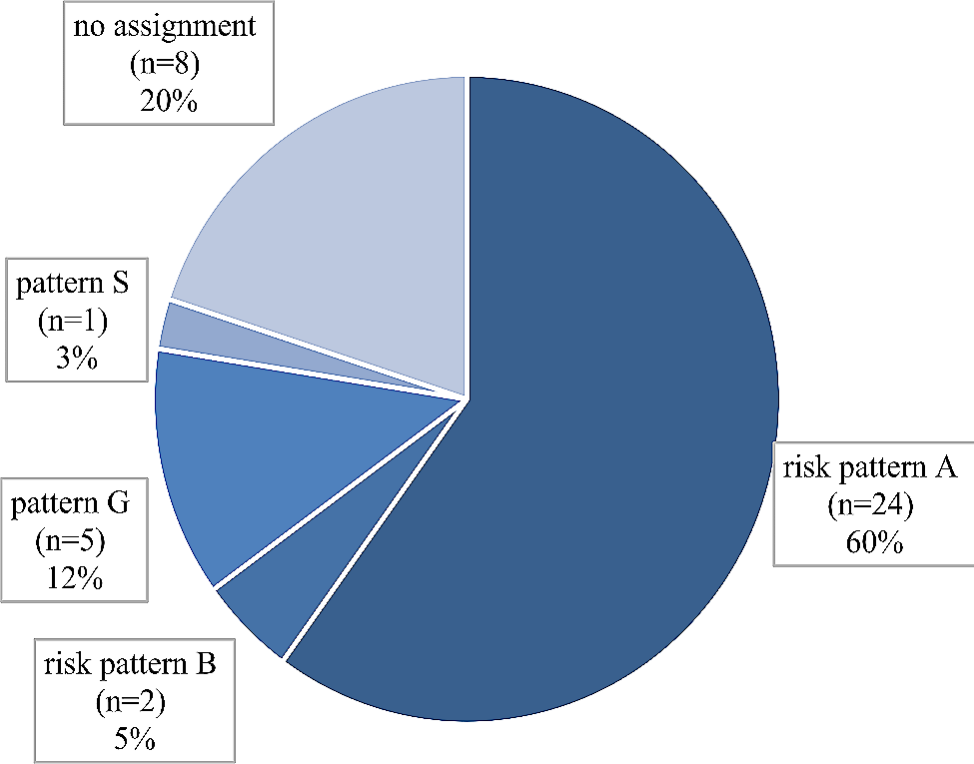
The distribution of AVEM patterns in the total sample

With regard to the total sample, 60% of the oncologists exhibited risk pattern A, and 5% exhibited risk pattern B (Fig. 1). A total of 12.5% of the respondents exhibited pattern G, while 2.5% exhibited pattern S. A clear assignment to a specific AVEM pattern or the combined patterns was not possible for 20% of the oncologists.

Risk patterns A and B predominated in the total sample (Table 2). The pattern distribution did not significantly differ between male and female oncologists (*p* = 0.830). Female oncologists were most commonly associated with risk pattern A (59.1%) and risk pattern B (4.5%), which were similar to the frequencies reported among their male colleagues (61.1% pattern A and 5.6% pattern B).

**Table 2:** Absolute and relative frequency of AVEM patterns by expression and gender

AVEM Most likely type	(Percentage) Frequency	Men *n* = 18	Women *n* = 22	Total *n* = 40	p_Pearson Chi²_
No assignment	Quantity	3	5	8	0.830
	% of AVEM type	37.5%	62.5%	100%	
	% from gender	16.7%	22.7%	20%	
	% of total	7.5%	12.5%	20%	
A	Quantity	11	13	24	
	% of AVEM type	45.8%	54.2%	100%	
	% from gender	61.1%	59.1%	60%	
	% of total	27.5%	32.5%	60%	
B	Quantity	1	1	2	
	% of AVEM type	50%	50%	100%	
	% from gender	5.6%	4.5%	5%	
	% of total	2.5%	2.5%	5%	
G	Quantity	3	2	5	
	% of AVEM type	60%	40%	100%	
	% from gender	16.7%	9.1%	12.5%	
	% of total	7.5%	5.0%	12.5%	
S	Quantity	0	1	1	
	% of AVEM type	0%	100%	100%	
	% from gender	0%	4.5%	2.5%	
	% of total	0%	2.5%	2.5%	
Total	Quantity	18	22	40	
	% of AVEM type	45%	55%	100%	
	% from gender	100%	100%	100%	
	% of total	45%	55%	100%	

### Results of the Differential stress inventory

The mean values and other descriptive statistics of the DSI dimensions are shown in Table 3. A relatively low level of the main variable, i.e., palliative coping, indicates that, in this overall sample, very few strategies are available to enable respondents to cope with the stress they face. This variable measures the availability of strategies that enable individuals to cope with stress through positive emotions and cognitions. Oncologists are not skilled at persuading themselves. They cannot offer sufficient positive instructions to themselves. The availability of instrumental coping strategies that enable people to cope with stress by actively addressing the cause of stress was also relatively low in this sample. The degree of external stress stabilization among oncologists was very pronounced (7.19 ± 2.098 stanine points). The risk in this context is that stress comes to be viewed as unavoidable and that a feeling of helplessness emerges. As a result, the individual’s ability to address future stress is weakened. The main variable of internal stress stabilization was very low in this sample (1.46 ± 0.948 stanine points). No risk of stress being viewed as the result of one’s own mistakes was observed in this context. This result is positive because self-blame, which further weakens the individual’s ability to deal with future stress, was absent in this sample.

The distributions of the DSI dimensions were identical between genders. A marginally significant finding (*p* = 0.085) indicated that male oncologists obtained higher scores with respect to instrumental coping and emotional-cognitive manifestations than did female oncologists.

**Table 3:** The expression of the DSI dimensions in the total sample and in both gender groups

DSI		Men *n* = 14	Women *n* = 12	Total *n* = 26	p_Mann−Whitney_
		AV ± SD [points] median (min - max) 95% confidence interval			
Causes of stress		5.21 ± 2.723 6 (1–9) [3.64–6.79]	3.67 ± 2.674 3.5 (1–9) [1.97–5.37]	4.50 ± 2.760 4.5 (1–9)	0.160
through	everyday events	4.93 ± 2.645 5.50 (1–9) [3.40–6.46]	3.75 ± 2.598 4 (1–8) [2.10–5.40]	4.38 ± 2.639 4(1–9)	0.322
	anxieties about life circumstances	5.07 ± 2.018 5.5 (2–9) [3.91–6.24]	4.25 ± 2.417 4 (1–9) [2.71–5.79]	4.69 ± 2.205 4.00 (1–9)	0.347
	interaction with others	5.14 ± 2.770 6 (1–9) [3.54–6.74]	3.92 ± 2.937 3.5 (1–9) [2.05–5.78]	4.58 ± 2.859 5.50 (1–9)	0.212
Coping		3.93 ± 2.433 4 (1–8) [2.52–5.33]	2.75 ± 2.598 1 (1–8) [1.10–4.40]	3.38 ± 2.531 3.00 (1–8)	0.231
through	instrumental	4.79 ± 2.359 5 (1–9) [3.42–6.15]	3.08 ± 2.644 2 (1–9) [1.40–4.76]	4.00 ± 2.592 4.00 (1–9)	0.085
	palliative	3.43 ± 2.277 3 (1–8) [2.11–4.74]	2.58 ± 2.193 1.5 (1–7) [1.19–3.98]	3.04 ± 2.236 2.50 (1–8)	0.274
Stress manifestation		4.79 ± 2.045 5 (2–8) [3.61–5.97]	3.58 ± 2.575 2.5 (1–9) [1.95–5.22]	4.23 ± 2.338 3.50 (1–9)	0.118
through	emotional-cognitive manifestation	4.64 ± 2.098 5 (1–9) [3.43–5.85]	3.17 ± 2.552 2.5 (1–9) [1.54–4.79]	3.96 ± 2.391 3.50 (1–9)	0.085
	physical manifestation	4.93 ± 1.685 5 (2–7) [3.96–5.90]	4.08 ± 2.314 3.5 (1–8) [2.61–5.55]	4.54 ± 2.005 4.00 (1–8)	0.297
Stress stabilization		4.43 ± 2.441 6 (1–7) [3.02–5.84]	3.08 ± 2.746 1 (1–8) [1.34–4.83]	3.81 ± 2.623 4.50 (1–8)	0.231
through	external	7.86 ± 1.791 9 (4–9) [6.82–8.89]	6.42 ± 2.234 6 (4–9) [5.00–7.84]	7.19 ± 2.098 8.50 (4–9)	0.106
	internal	1.29 ± 0.611 1 (1–3) [0.93–1.64]	1.67 ± 1.231 1 (1–4) [0.88–2.45]	1.46 ± 0.948 1.00 (1–4)	0.742

### Correlation analysis of the AVEM and DSI dimensions

Table 4*presents an overview of the correlation analysis.* Palliative coping was correlated with striving for perfection (ρ = 0.404 at *p* = 0.040), which was in turn positively related to external intensifiers (ρ = 0.439 at *p* = 0.025). In both cases, medium effect sizes were observed. Internal intensifiers were negatively correlated with the AVEM dimension of satisfaction with life (ρ = -0.504 at *p* = 0.009), and this effect was strong.

**Table 4:** correlation analysis of AVEM and DSI dimensions

			AVEM										
			Subjective importance of work	Work-related ambition	Willingness to work until exhausted	Striving for perfection	Distancing ability	Tendency to resignation in the face of failure	Proactive problem-solving	Inner calm and balance	Experience of success at work	Satisfaction with life	Experience of social support
*DSI*	Causes of stress		*0.010* *(0.961)*	*0.058* *(0.779)*	*0.195* *(0.341)*	*0.283* *(0.161)*	*-0.063* *(0.759)*	*-0.020* *(0.923)*	*-0.129* *(0.530)*	*-0.214* *(0.295)*	*0.157* *(0.442)*	*-0.268* *(0.185)*	*0.048* *(0.814)*
	through	everyday events	*0.112* *(0.587)*	*0.099* *(0.631)*	*0.191* *(0.349)*	*0.288* *(0.153)*	*-0.086* *(0.677)*	*-0.019* *(0.927)*	*-0.111* *(0.589)*	*-0.120* *(0.559)*	*0.205* *(0.315)*	*-0.272* *(0.178)*	*-0.028* *(0.893)*
		anxieties about life circumstances	*0.133* *(0.518)*	*0.202* *(0.321)*	*0.201* *(0.326)*	*0.306* *(0.129)*	*-0.252* *(0.214)*	*-0.017* *(0.934)*	*-0.145* *(0.481)*	*-0.244* *(0.229)*	*0.119* *(0.562)*	*-0.309* *(0.125)*	*0.138* *(0.501)*
		interaction with others	*0.007* *(0.974)*	*0.009* *(0.966)*	*0.185* *(0.366)*	*0.292* *(0.148)*	*-0.149* *(0.467)*	*-0.022* *(0.915)*	*-0.150* *(0.466)*	*-0.200* *(0.328)*	*0.082* *(0.692)*	*-0.316* *(0.116)*	*0.108* *(0.600)*
	Coping		*0.205* *(0.314)*	*0.079* *(0.702)*	*0.006* *(0.976)*	*0.296* *(0.142)*	*0.038* *(0.852)*	*-0.187* *(0.362)*	*0.008* *(0.970)*	*0.032* *(0.878)*	*0.111* *(0.589)*	*-0.340* *(0.089)*	*-0.070* *(0.733)*
	through	intrumental	*0.269* *(0.183)*	*0.142* *(0.490)*	*0.123* *(0.550)*	*0.310* *(0.124)*	*-0.037* *(0.857)*	*0.021* *(0.919)*	*-0.008* *(0.968)*	*0.004* *(0.984)*	*0.203* *(0.320)*	*-0.314* *(0.119)*	*-0.135* *(0.510)*
		palliative	*0.183* *(0.372)*	*0.066* *(0.750)*	*0.053* *(0.797)*	*0.404 (0.040)*	*0.008* *(0.970)*	*-0.263* *(0.195)*	*0.105* *(0.610)*	*0.015* *(0.944)*	*0.103* *(0.617)*	*-0.292* *(0.148)*	*-0.008* *(0.971)*
	Stress-manifestation		*0.130* *(0.525)*	*0.131* *(0.525)*	*0.303* *(0.132)*	*0.233* *(0.251)*	*-0.167* *(0.414)*	*0.071* *(0.731)*	*-0.049* *(0.811)*	*-0.212* *(0.297)*	*0.245* *(0.228)*	*-0.200* *(0.328)*	*0.099* *(0.630)*
	through	emotional-cognitive manifestation	*0.181* *(0.377)*	*0.121* *(0.555)*	*0.307* *(0.128)*	*0.218* *(0.284)*	*-0.199* *(0.330)*	*0.109* *(0.595)*	*-0.113* *(0.584)*	*-0.224* *(0.272)*	*0.178* *(0.384)*	*-0.208* *(0.307)*	*0.085* *(0.681)*
		physical manifestation	*0.019* *(0.925)*	*0.023* *(0.913)*	*0.358* *(0.072)*	*0.197* *(0.335)*	*-0.119* *(0.562)*	*0.087* *(0.672)*	*0.010* *(0.960)*	*-0.198* *(0.332)*	*0.204* *(0.318)*	*-0.266* *(0.189)*	*0.082* *(0.689)*
	Stress stabilization		*0.119* *(0.562)*	*0.053* *(0.798)*	*0.241* *(0.236)*	*0.356* *(0.075)*	*-0.077* *(0.710)*	*-0.080* *(0.697)*	*-0.016* *(0.937)*	*-0.105* *(0.610)*	*0.192* *(0.346)*	*-0.347* *(0.082)*	*0.008* *(0.967)*
	through	external	*0.204* *(0.319)*	*0.067* *(0.744)*	*0.109* *(0.597)*	0.439 (0.025)	*-0.020* *(0.922)*	*-0.154* *(0.452)*	*0.112* *(0.584)*	*-0.034* *(0.869)*	*0.227* *(0.264)*	*-0.254* *(0.210)*	*-0.062* *(0.762)*
		internal	*-0.078* *(0.706)*	*-0.107* *(0.604)*	*0.154* *(0.451)*	*-0.082* *(0.689)*	*-0.149* *(0.468)*	*0.077* *(0.708)*	*-0.188* *(0.358)*	*-0.261* *(0.197)*	*-0.280* *(0.165)*	-0.504 (0.009)	*-0.044* *(0.829)*
**Notes.** Spearman-Rho (ρ), *** the correlation is significant at the 0.001 level (two-sided); ** correlation at the 0.01 level; * correlation at the 0.05 level. Green = positive correlation, red = negative correlation. The stronger the color intensity, the stronger the effect. The effects of Spearman’s Rho (ρ): < 0.1 no effect, ρ = 0.1–0.29 weak effect, ρ = 0.300 to 0.499 medium effect and ρ ≥ 0.500 strong effect. AVEM = Work-related behavior and experience pattern DSI = Differential Stress Inventory													

## Discussion

The SARS-CoV-2 pandemic has had major impacts on job demands, job resources, employee outcomes and the ways in which employees perform their jobs [24]. Despite the recent crises in Ukraine, including the SARS-CoV-2 pandemic and the ongoing military conflict, oncologists working in Kharkiv have continued to perform their work, despite the effects of these crises on their own lives, the lives of their family members, and the lives of their patients. An additional challenge in this context has been the increasing absence of staff due to waves of doctors fleeing the country. A cross-sectional study cannot determine whether these crises weakened the health resources available to the surveyed physicians. The percentage of oncologists of both genders who exhibited risk patterns A and B at the time of the survey (mid-year 2022) was quite high (65%). This value is similar to the value reported for Ukrainian university lecturers at the Medical University, who already exhibited AVEM risk patterns of 64.8% before the emergence of the listed crises [25]. The AVEM values therefore indicate very high values with regard to the prevalence of risk patterns of work-related behavior and experiences in response to professional demands. The gender differences could only be viewed as a trend because the sample size was too small. Subsequently, German occupational groups exhibit various AVEM risk patterns, including rates of 32% among emergency medical services personnel [26], 34% among police officers [27], 40% among hospital physicians [27], 47% among psychotherapists [28], 41% among nurses [29] and 34% among bank employees (Voltmer et al. 2018). In all these occupational groups from Germany, respondents who exhibited risk pattern A or B were much less prevalent.

### Discussing work-related behavior and experience patterns and dimensions

The gender comparison did not reveal any meaningful differences in terms of the distributions of the patterns. This difference is likely related to the medical profession or the specialty of oncology, as female and male oncologists did not exhibit differences in terms of their coping patterns or ways of addressing stressful situations. In the literature, gender differences in AVEM expressions have been observed among teachers, with no fundamental shifts observed in the pattern distribution [30]. These four characteristic expressions are associated with mental health on the one hand and with health risks to the person in question on the other hand. To make cross-occupational statements, larger samples of different occupational groups (teachers, nurses, executives, senior administrators, people starting their own businesses, and firefighters) were included in a cluster analysis [21, 30–34]. Strong differences were observed in terms of the distribution of the patterns. The most favorable distribution was observed among executives from the Viennese health care system: risk patterns A and B accounted for only 13% of the total. Teachers were associated the worst patterns among occupational groups: the sum of the risk patterns was clearly the highest in this context. Schaarschmidt and Fischer showed that occupational conditions influence the pattern distribution. The distributions of risk patterns observed in this context allow us to draw conclusions concerning the overall situation of Ukrainian oncology during this crisis period [30]. Based on this study, it cannot be determined whether this situation is due to the quality of the job characteristics, framework conditions, job-related requirements, or stress associated with oncology or whether it is due instead to the stressful situation resulting from the pandemic and/or military conflicts and multiple additional stresses. Increased emotional and psychosocial stress is strongly suspected in this context.

The gender comparison did not indicate any significant differences in any of the dimensions, with the exception of the dimension of “experience of social support”. In this context, female oncologists rated their experience of social support more highly than did male oncologists. Information regarding social support, especially family support, outside the workplace as well a perceived social support from colleagues and superiors was included in this dimension, thus allowing us to obtain a more complete overview of this situation. This dimension included questions such as “My partner is understanding of my work”, “My family shows little interest in my work problems”, “I would like my partner to be more considerate of my work tasks and problems”, “I receive all the support I need from my family”, “Sometimes I would like more support from the people around me” and “If I ever need advice and help, there is always someone there”.

The dimensions of success at work, satisfaction with life, and social support are associated with the area of “emotions”. The latter dimension is regarded as a “psychological protective factor” with regard to critical situations but also as a direct expression of well-being and thus of mental health.

This occupational group exhibits higher values with regard to the dimensions of work engagement (work-related ambition, subjective importance of work, willingness to work until exhausted and striving for perfection). The working group associated with Schaarschmidt classified work engagement in relation to work stress as one of the most important mental aspects of health [35]. A measured and goal-oriented use of energy that is in line with personal priorities proves to be favorable in this context. Accordingly, high levels of the dimension’s subjective importance of work and work-related ambition must be accompanied by a clear—but not necessarily extraordinary—willingness to expend energy.

The AVEM domain of “resilience”, which includes the dimensions of the tendency to resign in the face of failure, proactive problem solving, and inner calmness/balance, was similar across the two genders and identified as an expression of and prerequisite for health. Researchers have long known that various psychological concepts play major roles in mental and cardiovascular health through people’s subjective perceptions of their problem-solving ability [17, 36–41].

### Discussing the Differential stress inventory

No gender differences were observed in terms of stress triggers, stress manifestations, available coping strategies, or risks of stress stabilization among Ukrainian oncologists. The relatively low level of palliative coping observed in this overall sample indicated that very few strategies are available to cope with existing stress. Strategies that enable individuals to cope with stress through positive emotions and cognitions are insufficient in this context. Ukrainian oncologists are not skilled at persuading themselves. This situation may be related to the lack of prospects to address the crisis. External stress stabilization is very pronounced among oncologists. In terms of preventative medicine, it is important to note that a risk may be present in this context because stress is viewed as unavoidable, and a feeling of helplessness thus emerges. It is important to strengthen individuals’ ability to deal with future stress. Internal stress stabilization among Ukrainian oncologists is very low. With the exception of internal stress stabilization, men obtained higher scores than did women. Internal stress stabilization indicates the presence of internal reinforcers that may lead to chronic stress. A higher score on this dimension indicates that the individual’s own mistakes are regarded as self-inflicted. Although no significant differences were observed in this context, higher values for the other DSI categories were also observed among men. It can therefore be argued that various events can trigger stress more easily in men. Likewise, existing stress can become manifest very strongly and lead to various symptoms of stress. In such cases, stress leads to mental and emotional preoccupation that can undermine the individual’s ability to act or take the form of physical symptoms such as pain or nausea [18]. Nonetheless, men also obtained higher scores on the instrumental and palliative coping dimensions; thus, it is possible that a balance between stress and perceived strain was evident in this context. This aspect includes talking oneself into a good mood, offering positive instructions or taking action to improve one’s situation [18]. A study of Ukrainian university professors revealed that the DSI categories of stress triggered by daily life, interaction, and existential anxiety had strong effects on the burnout dimension of emotional exhaustion. Medium-sized effects of stress triggers on the burnout dimension of cynicism/depersonalization were also observed [42]. Comparative international literature on DSI that includes data from other countries is not available in Pubmed.

### Discussing the correlation analysis of the AVEM and DSI dimensions

Only a few significant correlations between the AVEM and DSI dimensions were observed. The factor that exhibited the strongest negative correlation was satisfaction with life in the case of AVEM and internal stress stabilization in the context of DSI. As a result, life satisfaction leads to less stress stabilization, which could in turn lead to the chronification of stress. Internal stress stabilization is particularly high when mistakes are viewed as self-inflicted. High levels of internal stress stabilization weaken the individual’s ability to deal with future stress [18]. In this context, interventions such as coping and resilience training appear to be particularly suitable. The following are recommended as possible intervention measures: relaxation and compensation (e.g., through autogenic training, progressive muscle relaxation, breathing exercises) or acting out in the context of sports, gardening, or exercise with the goals of reducing stress and increasing inner restlessness and detachment. Similarly, promoting experiences of pleasure and contentment can counteract restricted feelings about life or general dissatisfaction. Realistic goal setting in the context of work tasks can counteract experiences of failure or tendencies toward resignation, and individual stress analysis and coping by learning short- and long-term coping strategies can lead to increasing feelings of relaxation. In terms of work organization, the promotion of team spirit and team mentality, the establishment of a positive work environment within the organization and the maintenance of social contacts, even during leisure time, can counteract the experience of insufficient social support.

Based on a salutogenetic approach, the personal protective factors associated with the ability to cope with the professional demands of oncology and interact with seriously ill cancer patients in a health-promoting way become more obvious. Strengthening resilience competencies also appears to make sense in this context. Greater resilience competency improves physician‒patient relationships and encourages physicians to remain in oncology settings without transitioning to a new specialty [43].

### Limitations

Overall, the sample referenced in this research was small; however, it was representative of oncologists in a large city in Ukraine. The questionnaire-based approach may have led to social desirability bias. The extent to which the crises themselves were triggers for the study results cannot be determined based on a cross-sectional study. All the data were collected based on self-reports by participants, which may have led to bias. Additional information concerning the physical health or emotional well-being of oncologists may be useful with regard to reducing response bias. The study did not investigate workloads or changes in workloads during the crises. The extent of active hostilities (including drone attacks, bombings, and the pandemic) at the time of data collection was not recorded. At the time, active fighting operations were ongoing in the city (including drone attacks, bombings, and the pandemic). The influence of these factors on the results could also be discussed in this context.

The study did not investigate why oncologists stayed in Ukraine. It can be assumed that these physicians were strongly attached to their clinic, team and medical ethos and therefore did not leave. Moreover, helping and treating civilian patients with malignant diseases is a high priority for physicians. In Syria, it more than 200,000 people are estimated to have died from cancer during the course of armed conflict due to a lack of adequate medical care [44]. Other examples of reasons why people are unable to leave their own country in the event of armed conflict include fear of the unknown (i.e., of not being welcome or of encountering difficulties while adapting to the new country), family bonds, financial reasons, fear of the inability to find work in a foreign country, and patriotism.

The crisis has changed job demands because all employees have been required to work differently as a result of the SARS-CoV-2 pandemic [24]. This crisis has disrupted work and organizations worldwide and increased threats to both employees’ own health and public health, thereby requiring additional effort on the part of employees [45]. This increase in work demands is not unique to the SARS-CoV-2 pandemic [24]. The other crisis also significantly changed the quality of work by increasing job complexity and leading to insufficient ergonomics [46] as well as by increasing uncertainty and insecurity [47].

## Conclusions

The lack of significant gender differences in the behaviors and coping styles exhibited by these oncologists allows gender-independent conclusions to be drawn. The results regarding Ukrainian oncologists presented here highlight some personal health resources on which they can draw when responding to stress in the situation with the SARS-CoV-2 pandemic and military conflicts in Ukraine and coping with daily professional demands.

The high prevalence of risk patterns observed in this research indicates that these resources are insufficient. For most respondents in this study, i.e., those who exhibited patterns A and B, the importance of interventions was highlighted from a health perspective. For respondents who exhibited behavior pattern G, such interventions were not found to be necessary.

## Data Availability

The data can be accessed via the corresponding author.

## Declarations

The survey was conducted in accordance with the requirements of bioethics approved at the meetings of the Committee on Ethics and Bioethics of Kharkiv National Medical University when planning the research (extract from Protocol No. 3 of August 28, 2020) and in accordance with the work plan for 2022 (extract from Protocol No. 3 of March 17, 2021).

## Abbreviations

A: AVEM risk pattern
AVEM: Work-Related Behavior and Experience Patterns (questionnaire)
B: AVEM risk pattern
DSI: Differential Stress Inventory (questionnaire)
G: AVEM pattern
Max: maximum
M: mean
Min: minimum
S: AVEM pattern
SD: standard deviation
